# *Mycoplasma hominis *brain abscess following uterus curettage: a case report

**DOI:** 10.1186/1752-1947-5-278

**Published:** 2011-07-03

**Authors:** Mouhamad Al Masalma, Michel Drancourt, Henry Dufour, Didier Raoult, Pierre-Edouard Fournier

**Affiliations:** 1 Federation de Microbiologie, Hôpital de la Timone, Marseille, France; 2 Service de Neurochirurgie, Hôpital de la Timone, Marseille, France

## Abstract

**Introduction:**

*Mycoplasma hominis *is mostly known for causing urogenital infections. However, it has rarely been described as an agent of brain abscess.

**Case presentation:**

We describe a case of *M. hominis *brain abscess in a 41-year-old Caucasian woman following uterus curettage. The diagnosis was obtained by 16S rDNA amplification, cloning and sequencing from the abscess pus, and confirmed by a specifically designed real-time polymerase chain reaction assay.

**Conclusions:**

Findings from our patient's case suggest that *M. hominis *should be considered as a potential agent of brain abscess, especially following uterine manipulation.

## Introduction

Brain abscess is a life-threatening condition resulting from the invasion of brain tissues by microorganisms. Current microbiological documentation, mostly based on direct examination and culture of pus specimens, may underestimate the role of fastidious microorganisms in brain abscess [1]. Among these, *Mycoplasma hominis *has rarely been reported [2-7]. *M. hominis *is a fastidious and slow-growing bacterium, commensal of the genitourinary tract of healthy adults. It mostly causes urogenital infections but may also cause extra-genital infections [8,9]. Infections caused by *Mycoplasma *sp. require specific antibiotic treatment. Lacking a cell wall and folic acid synthesis, they are resistant to antibiotics that target the cell wall or folic acid synthesis [10]. In particular, they are naturally resistant to β-lactams, which in combination with metronidazole have been recommended as empirical treatment of bacterial brain abscesses [11]. In contrast, *M. hominis *is sensitive to antibiotics that prevent the synthesis of proteins, including tetracyclines [12]. In addition, this bacterium cannot be Gram stained and requires specific culture media. However, molecular methods were successfully used to detect *M. hominis *from human samples [13].

## Case presentation

In 2006, a previously healthy, 41-year-old Caucasian pregnant woman was admitted to our hospital with vertigo, severe headache, and left hemiparesis. She had no relevant medical history except two previous normal pregnancies and deliveries. A computed tomography (CT) scan and MRI scan of the brain identified a right fronto-parietal hematoma. The hematoma was surgically drained. Then 10 days later, at 22 weeks of gestation, our patient underwent early spontaneous miscarriage that required uterus curettage, complicated by important metrorrhagia. At three days following the miscarriage, our patient developed obnubilation, and subsequently coma. New cerebral CT and MRI scans revealed a fronto-parietal brain abscess. The abscess was surgically removed, and purulent material was sent to our laboratory. A nosocomial infection being suspected, an intravenous empirical treatment associating vancomycin (2 g/day) and meropenem (6 g/day) was started. Gram staining of the abscess specimen showed numerous polymorphonuclear leukocytes but no microorganism. The specimen was then plated onto 5% sheep blood agar and chocolate agar (BioMérieux, Marcy L'Etoile, France) and incubated at 37°C under aerobic, anaerobic, and microaerophilic conditions for 10 days. Plates were examined daily but no growth was observed. For molecular detection, DNA was extracted from the pus sample using the MagNA Pure LC DNA isolation kit II and the MagNA Pure LC instrument as recommended by the manufacturer (Roche, Meylan, France). Amplification and sequencing of the 16S rDNA gene were performed using broad range primers as previously described [14]. By comparison with sequences from GenBank, the sequence obtained from the polymerase chain reaction (PCR) product (1,475 bp) was 100% identical to that of *M. hominis *(GenBank accession number AF443616). As a consequence, the antibiotic treatment was changed to doxycycline, 200 mg/day for 12 weeks. Our patient recovered rapidly. On follow-up, she remained asymptomatic six months after the discontinuation of antibiotics. In order to determine whether the infection was monomicrobial or polymicrobial, the PCR amplicon was subsequently cloned into *Escherichia coli *using the pGEM-T Easy Vector System (Promega, Charbonnières, France). A total of 100 clones were analyzed by sequencing. Only 16S rDNA from *M. hominis *was detected in the 100 clones. The identification of *M. hominis *in our patient and the previously published cases motivated the development of a specific real time-PCR (RT-PCR) assay for this bacterium. 16S rDNA was selected as target. Using the Primer Express software (Applied Biosystems), specific primers and probes were designed as follows: MHMGB16Sd (5'-TGT TAT AAG GGA AGA ACA TTT GCA AT-3'), MHMGB16Sr (5'-GCC ATC GCT TTC TGA CAA GG-3') and MHMGB16S probe (FAM-AAA-TGA-TTG-CAG-ACT-GAC-MGB) respectively. RT-PCR was performed using a LightCycler (Roche). The PCR mix consisted of 4 μL of pus DNA, 10 μL of Quantitect Probe PCR Master Mix (Qiagen, Courtaboeuf, France), 20 pM of each primer (Eurogentec, Seraing, Belgium), 0.5 μL of Uracil DNA glycosylase (Invitrogen), 0.5 μL of 3.125 μM MHMGB probe (Applera), and 4 μL of water. DNA was amplified using the following cycling parameters: heating at 50°C for 2 minutes, and then at 95°C for 15 minutes, followed by 50 cycles of a two-stage temperature profile of 95°C for one second and 60°C for 45 seconds. The specificity of the primers and probes was tested using BLAST http://blast.ncbi.nlm.nih.gov/ and by tentatively amplifying DNA from 24 distinct *Mycoplasma *species. The system was found to be specific *to M. hominis*, as no amplification was obtained from any other mycoplasmal or human DNA. For our patient, positive amplification was obtained after 22 PCR cycles. Negative controls remained negative.

## Discussion

*M. hominis *frequently colonizes the lower genitourinary tract of women [15]. Host predisposing factors such as immunosuppression, malignancy, trauma, and manipulation or surgery of the genitourinary tract are considered as risk factors of extra-genital infections. It was notably demonstrated that blood spread of mycoplasmas may follow urinary tract catheterization or lithiasis [16]. To the best of our knowledge, *M. hominis *has previously been reported in only six patients as a cause of brain abscess [2-7] (Table 1). In the three female patients, *M. hominis *infection complicated a traumatic or spontaneous brain hematoma in a context of normal vaginal or cesarean delivery [2,3,7]. In the two male adult patients, the *M. hominis *infection complicated a head trauma in the context of urinary tract catheterization [4,5]. In female patients, the most likely source of *M. hominis *was the genital tract whereas it was the urinary tract in men. The most recent patient, a three-week-old baby, most likely acquired the *M. hominis *infection from passage through the maternal birth canal [6]. In our patient, we assume that the source of infection was the genital tract, as our patient underwent uterine curettage. It should be noted that in most cases, *M. hominis *superinfected a brain hematoma. By searching the literature for other cases of *M. hominis *infection of hematomas, we found six articles describing patients who had developed infection of abdominal, peri-nephric, thigh or retroperitoneal hematomas following genitourinary invasive procedures [17-22] (Table 2). In an additional patient, infection complicated a peri-hepatic hematoma but the origin of infection was not identified [23]. Therefore, *M. hominis *appears to have a particular ability for superinfecting hematomas, in particular following genitourinary tract invasive procedures.

**Table 1:** Epidemioclinical features of previously reported patients with *Mycoplasma hominis *brain abscess

Sex/age	Medical history	Identification	Reference
M/29	Traumatic brain hematoma and urinary tract catheterization	Culture	[5]
M/40	Head trauma and urinary tract catheterization	PCR	[4]
F/22	Brain hematoma following normal vaginal delivery	Culture	[7]
F/17	Subdural hematoma following normal full term pregnancy and delivery	Culture	[2]
F/32	Subdural hematoma following cesarean delivery	Culture	[3]
M/3 weeks	Normal full term pregnancy and delivery	PCR	[6]

PCR = polymerase chain reaction.

**Table 2:** Cases of hematoma (other than brain) infected with *Mycoplasma hominis*

Sex/age	Medical history	Identification	Reference
F/27	Abdominal hematoma following cesarean section	Culture	[19]
F/27	Abdominal hematoma following cesarean section	Culture and PCR	[21]
M/74	Wound and peri-nephric hematoma following renal transplantation	Culture	[22]
F/18	Peri-nephric hematoma following renal transplantation	Culture	[20]
F/36	Thigh hematoma following trauma of pelvis and genitourinary tract	Culture	[18]
M/55	Peri-hepatic hematoma following liver transplantation	Culture	[23]
M/29	Retroperitoneal hematoma following pelvis trauma	Culture	[17]
F/69	Subcutaneous hematoma and respiratory tract infection	Culture	[24]

PCR = polymerase chain reaction.

In addition, as previously reported [4], bacterial culture and Gram staining results remained negative. *M. hominis *was only detected by PCR. In addition, in an effort to reduce the diagnostic delay, we developed a specific RT-PCR for *M. hominis*. This test provides a rapid alternative not only to culture but also to broad-range 16S rRNA PCR and sequencing detection, and may enable rapid antibiotic treatment adaptation.

## Conclusions

Our data suggest that *M. hominis *should be suspected in patients developing brain abscess following genitourinary tract invasive procedures, notably uterine curettage. To facilitate the detection of this agent, we developed an accurate, sensitive, and specific RT-PCR assay for *M. hominis *that may enable the diagnosis to be obtained within one hour of DNA extraction.

## Consent

Written informed consent was obtained from the patient for publication of this case report and any accompanying images. A copy of the written consent is available for review by the Editor-in-Chief of this journal.

## Competing interests

The authors declare that they have no competing interests.

## Authors' contributions

MAM and PEF wrote the manuscript while MD performed the microbiological identification. HD performed the surgical treatment and revised the manuscript. DR corrected the manuscript. All authors read and approved the final manuscript.
